# Potential Drug-Drug Interactions among Patients prescriptions collected from Medicine Out-patient Setting

**DOI:** 10.12669/pjms.341.13986

**Published:** 2018

**Authors:** Riffat Farooqui, Talea Hoor, Nasim Karim, Mehtab Muneer

**Affiliations:** 1 Dr. Riffat Farooqui, BDS, M. Phil. Associate Professor, Pharmacology Department, Bahria University Medical and Dental College Sailors Street, Adjacent PNS Shifa, Defence Phase -2, Karachi, Pakistan; 2 Dr. Talea Hoor, MBBS, M. Phil. Associate Professor, Pharmacology Department, Bahria University Medical and Dental College Sailors Street, Adjacent PNS Shifa, Defence Phase -2, Karachi, Pakistan; 3 Dr. Nasim Karim, MBBS, M. Phil, Ph.D, Post Doc (USA). Head of Pharmacology Department, Pharmacology Department, Bahria University Medical and Dental College Sailors Street, Adjacent PNS Shifa, Defence Phase -2, Karachi, Pakistan; 4 Dr. Mehtab Munir, MBBS. Senior Lecturer, Pharmacology Department, Bahria University Medical and Dental College Sailors Street, Adjacent PNS Shifa, Defence Phase -2, Karachi, Pakistan

**Keywords:** Rational prescribing, Potential drug-drug interactions, Frequency, Severity, Mechanism, Common pairs

## Abstract

**Objective:**

To identify and evaluate the frequency, severity, mechanism and common pairs of drug-drug interactions (DDIs) in prescriptions by consultants in medicine outpatient department.

**Methods:**

This cross sectional descriptive study was done by Pharmacology department of Bahria University Medical & Dental College (BUMDC) in medicine outpatient department (OPD) of a private hospital in Karachi from December 2015 to January 2016. A total of 220 prescriptions written by consultants were collected. Medications given with patient's diagnosis were recorded. Drugs were analyzed for interactions by utilizing Medscape drug interaction checker, drugs.com checker and stockley`s drug interactions index. Two hundred eleven prescriptions were selected while remaining were excluded from the study because of unavailability of the prescribed drugs in the drug interaction checkers.

**Results:**

In 211 prescriptions, two common diagnoses were diabetes mellitus (28.43%) and hypertension (27.96%). A total of 978 medications were given. Mean number of medications per prescription was 4.6. A total of 369 drug-drug interactions were identified in 211 prescriptions (175%). They were serious 4.33%, significant 66.12% and minor 29.53%. Pharmacokinetic and pharmacodynamic interactions were 37.94% and 51.21% respectively while 10.84% had unknown mechanism. Number wise common pairs of DDIs were Omeprazole-Losartan (S), Gabapentine- Acetaminophen (M), Losartan-Diclofenac (S).

**Conclusion:**

The frequency of DDIs is found to be too high in prescriptions of consultants from medicine OPD of a private hospital in Karachi. Significant drug-drug interactions were more and mostly caused by Pharmacodynamic mechanism. Number wise evaluation showed three common pairs of drugs involved in interactions.

## INTRODUCTION

When a patient presents with a medical problem and diagnosis is made the very next usual step is to advise drug therapy.^1^ Use of four or more medications (polypharmacy) found in prescriptions is common and is known to facilitate increase number of drug-drug interactions (DDIs).^2^ Intake of more than two drugs usually increases the risk of interaction between the drugs. The factors which considerably contribute to one or more interactions include: polypharmacy, patient`s age more than 60 years and those having cardiovascular diseases and other co-morbids.^3^ Thus concurrent administration of two or more drugs may results in decreased response or an increased risk of adverse reaction.^4^

Interaction between drugs may also results in favorable and unfavorable responses.^5^ The favorable response increases the effectiveness of drug, decreases the risk of adverse events and allows use of small doses while unfavorable response may increase the drug effectiveness but may cause unwanted even toxic effects in the body. Hence DDIs may increase or decrease the therapeutic effect or increases unwanted effects of many drugs.^6^ Tatro has documented that DDIs can be serious, significant and minor respectively depending upon their severity.^4^,^7^

An example of major/ severe/ serious DDI is combination of digoxin and spironolactone in which spironolactone decreases the clearance of digoxin and in this manner promotes digoxin toxicity.^8^ An example of moderate/ significant/ intermediate drug-drug interaction is combination of iron and pantoprazole in which the latter decreases gastric acidity and reduces bioavailability of the former.^9^ An example of minor/ mild/ less significant drug-drug interaction is the combination of aspirin and clopidogrel in which aspirin enhances the antiplatelet effect of clopidogrel and may results in major bleeding.^10^ If a drug-drug interaction is of serious domain then the combination should be avoided while in moderate type alternate drug for any of the two in combination should be used and lastly in case of minor drug-drug interaction the prescriber should remain vigilant thus to ensure safe use of drugs in patients.

On the basis of mechanism DDIs are divided into pharmacokinetic and pharmacodynamic drug-drug interactions. In pharmacokinetic DDIs plasma concentration of interacting drugs may be increased or decreased depending on the type of interaction on the other hand in pharmacodynamic DDIs interacting drugs either produce synergistic or antagonistic effects. A potentially significant interaction is one in which an unpredicted change in the state of the patient occur as a result of the use of therapeutic combination of drugs. DDI is a main causative factor for unwanted effects of drugs. Around 3-5% of all adverse drug reactions may results from drug-drug interactions.^11^

A significant percentage of adverse drug reactions are produced by drug-drug interactions.^12^ A range of 4.7% – 8.8%^13^ of DDIs has been observed in patients. So far majority of information sources are unable to mention significant interactions between drugs and include invalid, unrelated and inappropriate data.^14^-^16^ Physicians have special expertise, skills and professional judgment therefore they are expected to look up patient safety when prescribing drugs. In outpatient settings of tertiary care hospitals patients are subjected to use many drugs but knowledge about outpatients exposed to drug-drug interactions is inadequate.^17^ Drug-drug interactions are a patient related risk and danger to public health. In our clinical set-ups availability of such documented information is rare. Present study was designed to identify and evaluate the frequency, severity, mechanism and common pairs of DDI in prescriptions from Medicine Outpatient Department (OPD) of a private hospital in Karachi.

## METHODS

This cross sectional descriptive study was conducted after approval from the Research Review Committee and Ethical Review Committee of Bahria University Medical and Dental College as a part of main project “Prescribing patterns in hospital inpatients”. Prescriptions of consultants were collected from the patients in the medicine outpatient department, of a private hospital in Karachi, after verbal informed consent.

Prescriptions were collected by visiting medicine outpatient setting (OPD) twice weekly for two months from 1^st^ December 2015 to 31^st^January 2016. Adult male and female patients whose prescription contained at least two drugs and who gave their consent, were included in the study while hospital inpatient, children, pregnant and lactating women, patients with terminal illnesses and those who did not gave consent were excluded from the study.

A total of 220 prescriptions were collected, out of which 211 were selected while 09 prescriptions were excluded because of unavailability of the prescribed drugs in the drug interaction checkers. Drugs generic names were obtained from Pharmaguide 20^th^ edition and internet sources. ATC classification system was used for the classification of drugs^18^ The severity of prescribed drugs interactions were analyzed by Medscape Drug Interaction Checker i.e. Serious, Significant and Minor and reconfirmed by drugs.com checker and stockley`s drug interactions index. Results are expressed as mean and percentage.

## RESULTS

Prescriptions of 220 patients were collected, of which 211 were analyzed. Out of 211 patients 28.43% were suffering from diabetes mellitus, 27.96% patients were found to have hypertension, 10.42% of patients were with generalized body ache (Table-I). Total numbers of drugs were 978 and average number of drugs per prescription was 4.6. All prescription had more than one medication, 32.22% prescriptions had four medications, 16.53% prescriptions had three medications and 15.63% prescriptions contained five medications (Table-I). 17.38% drugs belong to alimentary tract and metabolism, 16.35% belong to cardiovascular system, 16.15% belong to hormonal system and 15.84% belong to analgesics and antipyretics (Table-II). In present study 4.33% interactions were serious, 66.12% were significant and 29.53% were minor (Table-III). About 51.21% drug-drug interactions were at pharmacodynamic level and 37.94% were due to pharmacokinetic mechanisms (Table-III). The common drug pairs for serious, significant and minor drug interactions were identified (Table-IV).

**Table-I:** Diagnosis & Number of Medications Prescribed.

Diagnosis of Patients N=211	

Diseases	Number of Patients (%)
Diabetes	60(28.43%)
Hypertension	59(27.96%)
Generalized body ache	22(10.42%)
Gastritis	13(6.16%)
Asthma	07(3.31%)
Hypothyroidism	06(2.84%)
Hyperthyroidism	06(2.84%)
Acute Pharyngitis	06(2.84%)
Osteoarthritis	05(2.36%)
Rheumatoid arthritis	05(2.36%)
Tuberculosis	04(1.89%)
Others	18(8.53%)

Medications Prescribed N= 211	

Number of Medications	Number of Prescriptions

2	14 (6.63%)
3	35(16.53%)
4	68(32.22%)
5	33(15.63%)
6	29(13.74%)
7	23(10.90%)
8	8(3.79%)
9	1(0.47%)

N= number of prescriptions.

**Table-II:** System wise distribution of prescribed drugs. N= 978.

Therapeutic Groups	Medications
A Alimentary tract and metabolism	170(17.38%)
B Blood and blood forming organs	85(8.69%)
C Cardiovascular system	160(16.35%)
H Hormonal system	158(16.15%)
J Anti-infectives for use	45(4.60%)
N Nervous system	
Analgesics and antipyretics	155(15.84%)
Other nervous system drugs	45(4.60%)
R Respiratory system	44(4.49%)
Others	116(11.86%)

(As per ATC)^18^

**Table-III:** Intensity & mechanism of drug-drug interactions. N=369

Intensity	Percentage of drugdrug interactions	Mechanism	Percentage of drug-drug Interactions
Serious	16 (4.33%)	Pharmacokinetic (PK)	140(37.94%)
Significant	244 (66.12%)	Pharmacodynamic (PD)	189 (51.21%)
Minor	109 (29.53%)	Unknown	40 (10.84%)

**Table-IV:** Common pairs of drug interaction. N= 369

No	Serious	n=16	Significant	n=244	Minor	n=109
1	Diclofenac+ Methotrexate	4	Omeprazole+ Losartan	47*	Gabapentin+ Acetamiphen	22**
2	Celecoxib+ Methotrexate	2	Losartan+ Diclofenac	19***	Omeprazole+ Tizanidine	14
3	Fenofibrate+ Omerprazole	2	Diclofenac+ Glimepride	15	Hydrochlorthiazide+ Metformin	11

Most common*,

Second common**,

Third common***

## DISCUSSION

Prescribing appropriate drug(s) is the requirement of rational use of drugs.^19^ With the progression of enormous electronic primary care data sets, there is the choice to describe the population, its demography, co-morbidity, and recent prescribing.^20^,^21^ Drug interactions and their consistent adverse effects are the major reasons of hospital admission and mortalities. Outcomes resulting from the drugs account for approximately 10 to 20% of admissions to the hospital furthermore 1% hospital admissions result from DDIs.^22^ In a study conducted in medicine department of a tertiary care hospital the mean number of drugs per prescription was 7.8 per patient.^23^ Multiple drug therapy is usually required to treat patients with diabetes mellitus. Several drugs in a prescription and the event resulting from drugs are caused by the use of blood glucose lowering agents along with other drugs required to treat coexisting ailments.^24^

In our study mean number of drugs per prescription was 4.6 and majority of them (28.43) were suffering from diabetes mellitus. A study has documented significant drug-drug interactions in 92.8% of patients with diabetes mellitus.^25^ Drugs acting on alimentary tract and metabolism, cardiovascular system and hormonal system were the most prescribed drugs 17.38%, 16.35% and 16.15% respectively. These results are found to be in accordance with the study by Kaliamurthy where 140 patients were on multiple drug therapy. Anti-hypertensive and oral hypoglycemic drugs were the most frequently used drugs.^2^ Drugs acting on cardiovascular system (CVS) has also implicated in DDI as highlighted by several studies.^26^,^27^ Our study was centered around patients of outpatient department and this might be the reason that cardiovascular drugs are not at the top of the list. In present study total number of potential drug- drug interactions identified was 369. Out of these 4.33% were serious, 66.12% were significant and 29.53% were minor interactions. About 51.21% of the interactions were caused by pharmacodynamic mechanism and 37.94% of interactions were at pharmacokinetic level. This was similar to the study in which 390 interactions were found. Among them, majority of the interactions were of moderate severity (n=257, 65.9%), followed by minor interactions (n=120, 30.77%). About 51.8% were due to pharmacodynamic interactions while 48.2% were caused by pharmacokinetic interactions.^2^ In our study frequently occurring pair of drug-drug interaction were Omeprazole- Losartan, (47-S), Gabapentin-Acetaminophen (22-M), Losartan-Diclofenac (19-S) and Diclofenac-methotrexate (4-SE). It is known that in the combination of omeprazole and losartan, omeprazole acts as an inhibitor of hepatic drug metabolizing enzyme and increases the level and effect of losartan. It is also known that gabapentin reduces the blood concentration of acetaminophen by increasing its metabolism. Diclofenac decreases the antihypertensive effect of losartan by opposing drug effects. More over a serious interaction may occur if combination of Diclofenac and methotrexate is used. Diclofenac may elevate the concentration of methotrexate by reducing its clearance through the kidneys.^28^

### Limitations of the study

The present study had some limitations as Medscape was the principle source of drug interaction information. Since this study was a cross sectional study the estimation of adherence to the drug therapy by patients is not ascertained neither actual outcome of DDIs is evaluated. Studies, with large sample size, using more DDIs information sources, follow up of patients to record definite consequences and actual outcome are open avenues for future research.

## CONCLUSION

The frequency of DDIs in prescriptions by consultants from medicine outpatient department of a private hospital in Karachi is high. Majority of interactions were found to be of significant severity occurring at pharmacodynamic level.

## RECOMMENDATIONS

The safest approach to avoid potential DDIs is the implementation of appropriate guidelines, detailed and rationalized knowledge of drugs known to interact along with computer based screening will help the health care professionals to detect and reduces the chances of DDIs. Awareness should be disseminated through print and electronic media to the physicians, consultants and health care professionals. Continuous Medical Education sessions (CME`s) should be conducted and should be accessible by the health care professional to boost up their knowledge on regular basis with emphasis on rational use of drugs. Drug-drug interaction should also be included in the curriculum of undergraduate medical and dental students. Pharmacists should be included as member of the healthcare team to raise the standard of rational prescribing and ensuring patient safety.

### Authors' Contribution

**RF:** Conducted research and did main write up of manuscript

**TH:** Helped in data collection, writing and proof reading of manuscript

**NK:** Perceived, designed, revised critically for important intellectual content and is supervisor of the study.

**MM:** Helped in Data Analysis.
